# Antiviral Activity of Carrageenans and Processing Implications

**DOI:** 10.3390/md19080437

**Published:** 2021-07-30

**Authors:** Milena Álvarez-Viñas, Sandra Souto, Noelia Flórez-Fernández, Maria Dolores Torres, Isabel Bandín, Herminia Domínguez

**Affiliations:** 1 CINBIO, Faculty of Science, Universidade de Vigo, Campus Ourense, As Lagoas, 32004 Ourense, Spain; milalvarez@uvigo.es (M.Á.-V.); noelia.florez@uvigo.es (N.F.-F.); matorres@uvigo.es (M.D.T.); 2 Departamento de Microbioloxía e Parasitoloxía, Instituto de Acuicultura, Universidade de Santiago de Compostela, 15782 Santiago de Compostela, Spain; sandra.souto@usc.es (S.S.); isabel.bandin@usc.es (I.B.)

**Keywords:** sulfated polysaccharides, red seaweed, extraction, depolymerization

## Abstract

Carrageenan and carrageenan oligosaccharides are red seaweed sulfated carbohydrates with well-known antiviral properties, mainly through the blocking of the viral attachment stage. They also exhibit other interesting biological properties and can be used to prepare different drug delivery systems for controlled administration. The most active forms are λ-, ι-, and κ-carrageenans, the degree and sulfation position being determined in their properties. They can be obtained from sustainable worldwide available resources and the influence of manufacturing on composition, structure, and antiviral properties should be considered. This review presents a survey of the antiviral properties of carrageenan in relation to the processing conditions, particularly those assisted by intensification technologies during the extraction stage, and discusses the possibility of further chemical modifications.

## 1. Introduction

Novel antivirals are demanded to fight against viral infections with no available treatment but also to overcome certain problems associated with the use of some current drugs, such as unsatisfactory efficacy, adverse effects, toxicity, viral resistance, and high cost [1,2,3,4,5,6,7].

Sulfated polysaccharides mimicking the heparan sulfate are potential antivirals that can interfere with the early stages of viral replication, including the virus entry, by masking the positive charge of the virus surface receptors to prevent them from binding to the heparan sulfate proteoglycans in the host cell surface [2,8,9,10,11]. Although highly sulfated synthetic glycomimetics are effective inhibitors of viral infection both in vitro and in vivo [12], natural polysaccharides from renewable sources offer advantages in relation to their low cost, absence of toxicity and side effects, wide spectrum of activity, and low induction of resistance [13,14]. Recent research on the strong antiviral activity of polysaccharides, particularly against enveloped viruses, can be found [9,10,14].

Seaweeds are attractive candidates for developing potential antivirals due to their relatively high content of sulfated polysaccharides, low production costs, and wide availability; they are biodegradable, biocompatible, safe, and non-toxic [2,15,16,17]. The structural diversity and complexity of seaweed polysaccharides and their derivatives contribute to their activity in different phases of viral infection processes. In addition, several sulfated seaweed polysaccharides were found as immunomodulators [18,19] and can be used as vaccine adjuvants in nanomaterials and drug delivery systems [17].

Red seaweed polysaccharides contain different sulfated galactans, sulfated mannans, carrageenans, and agars, which are active against a wide range of viruses [20]. Sulfated galactans are the main matrix polysaccharide, found as agar or as carrageenan [20,21]. Carrageenans are approved as food grade additives and are used as thickeners and emulsifiers. In addition to the various biological activities such as anti-thrombotic, antiviral, anticancer, and immunomodulatory properties, carrageenan can be used to prepare microdimensional-based structures, useful for easy and controlled drug administration [22].

The biological activity of these high-molecular-weight compounds is determined by their chemical structure including the degree of sulfation, molecular weight, constituent sugars, and their stereochemistry [23]. Despite having good inhibitory effects on virus replication, the high-molecular-weight (MW)-associated poor tissue-penetrating ability of sulfated polysaccharides limits their potential antiviral application in humans. Therefore, carrageenan and its depolymerized products, oligosaccharides, are considered a good alternative for the prevention of a wide range of diseases, mainly caused by enveloped viruses [13,24,25].

Different authors have reviewed the antiviral activities of marine-derived polysaccharides, low-molecular-weight fractions and derivatives, and their underlying mechanisms of action and medical applications [2,26,27,28,29]. Ngo and Kim focused on sulfate polysaccharides derived from marine algae and presented an overview of their biological activities, including antiviral [18]. Besednova and co-authors reviewed the studies on the potential use of different algal metabolites, including polysaccharides, for the prevention and treatment of human immunodeficiency virus (HIV) [16]. Pagarete and coworkers [30] presented a survey on algal antivirals. The dietary and pharmaceutical applications of polysaccharides in the prevention and treatment of viral diseases [31] and the marine glycan-based antiviral drugs undergoing preclinical and clinical trials [2] have been recently reviewed. Lee presented a complete overview of the potential of carrageenans as preventive microbicides, based on in vitro, in vivo, and ex vivo assays of activity and safety [32]. Cheong and co-authors published a new perspective on the potential applications of carrageenan oligomers in the functional food and pharmaceutical industry with an emphasis on the production, purification, analysis, characterization, and biological properties [33]. The COVID-19 pandemic, caused by the SARS-CoV-2 virus, caused some researchers to reconsider the potential of algal metabolites [3,34,35], particularly from seaweeds [36,37]. Chen and co-authors reviewed the antiviral properties, mechanisms, and applications of some polysaccharides as therapeutic agents and vaccine adjuvants for the treatment of coronavirus [17].

The possibility of obtaining these natural compounds in large amounts from different algae converts the extraction and depolymerization processes in key aspects, affecting the structure and activity. The present review discusses the relevance of the processing conditions on the antiviral properties of carrageenans.

## 2. Carrageenans

**Figure 1:**
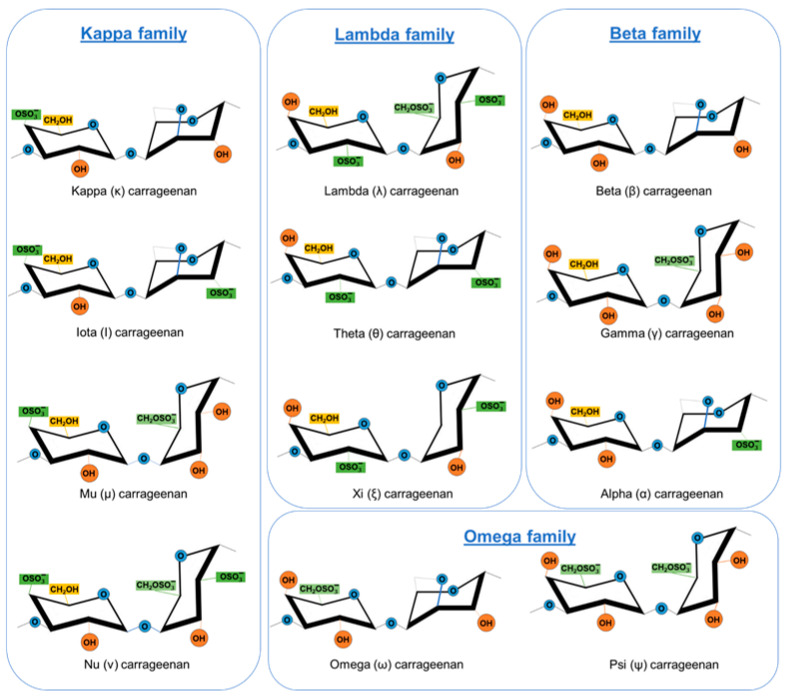
Basic carrageenan forms: iota (ι), kappa (κ), lambda (λ), mu (μ), nu (ν), theta (θ), xi (ξ), alpha (α), beta (β), gamma (γ), omega (ω), and psi (Ψ).

Carrageenans consist of sulfated linear polysaccharides of alternating (1→3)-β-d-galactopyranoses and (1→4)-α-d-galactopyranoses (or 3,6-anhydrogalactopyranoses), substituted with sulfate esters in different positions [38]. They are classified into various types named by Greek letters (iota, kappa, lambda, or mu) according to their structural characteristics, the sulfation patterns, and the presence or absence of 3,6-anhydro bridges in α-linked galactose residues (Figure 1). Lambda-carrageenans are formed by galactose units, whereas iota- and kappa- contain equal amounts of galactose and 3,6 anhydrogalactose. Kappa-carrageenan contains one sulfate group, iota-carrageenan contains two per disaccharide at axial positions, and lambda- has close to three equatorial sulfates. Natural carrageenans usually occur as mixtures of different hybrid types, such as κ/ι-hybrids, κ/μ-hybrids, or μ/ι-hybrids, which often form cyclized derivatives. Kappa- and iota-carrageenans frequently contain variable amounts of their biological precursors, namely mu- and nu-carrageenans, respectively [39]. The three most commercially exploited carrageenans are κ-, ι-, and λ-carrageenans [40]. Carrageenan structures differ among species of red algae and may also occur among the life stages of the same species [41]; even different composition was observed depending on the part of the alga, as reported for carrageenan κ-2 type, found in female gametophyte and λ-type in tetrasporophyte from *Gigartina atropurpurea* [23]. Carrageenans are found in red seaweeds, accounting for 30–75% of their dry weight (Table 1).

No toxicity was detected for different carrageenan types, isolated from a variety of algae and algal forms (tetrasporic/cystosporic) [23,42], or for chemically modified carrageenans [43]. In many cases, carrageenan treatment does not show harmful effects on metabolic activity, and the absence of morphological alterations in different cells at low concentrations was confirmed, but at higher concentrations, they can reduce the metabolic activity [1,8,24,39,44]. Absence of irritation or toxicity was also observed in in vivo assays [44]. From a recent compilation of safety profiles for different types of carrageenans in subclinical assays with different cells and in clinical studies [32], half maximum cytotoxicity concentrations were in the range 5–3000 µg/mL with a slight trend showing lower toxicity for the more sulfated carrageenans. Similarly, the λ-carrageenan show low toxicity in comparison to κ- and ι- carrageenans [13,45,46,47]. Native non-degraded carrageenan is non-toxic [48] and is a food additive (E-407) approved by the European Food Safety Authority and a natural ingredient, generally recognized as safe (GRAS) by the Food and Drug Administration.

Experiments in animals for the evaluation of the safety of intranasal administration of ι-carrageenan have provided no evidence of toxicity or intolerance when the polymer was applied intranasally or by inhalation [59]. No evidence of toxicity was found in a study on the exposure of neonatal pig to high doses of κ/λ-carrageenans with molecular weight of 664–732 kDa [60]. No toxicity in mice was detected in the study of the antiviral activity of *Gigartina skottsbergii* carrageenans against intraperitoneal murine herpes simplex virus infection [43]. A recent comprehensive review [32], also indicated that most of in vitro carrageenan studies demonstrated their superior cytotoxicity profiles (the highest CC_50_ values surpassed 1000 μg/mL), which seems to be an unattainable concentration in vivo applications. No toxicity of λ-carrageenans to the host cells was observed at concentrations up to 300 μg/mL. These carrageenans purified from marine red algae exhibited antiviral activity against influenza viruses and severe acute respiratory syndrome coronavirus 2 [61].

However, products of the acid hydrolysis of carrageenans, which present lower molecular “degraded carrageenans”, have been reported to be able to induce gastrointestinal irritation and cancer in animal models [48]. Oligosaccharides derived from carrageenans have been reported to present toxicity [40], and prudential use has been recommended in spite of their promising biological activities.

## 3. Mechanisms of Antiviral Activity

Viruses can be divided into two main categories: enveloped viruses, which have a lipid membrane (envelope) that is derived from the host cell, and nonenveloped viruses, which lack a membrane. The presence/absence of the envelope may condition their susceptibility to different disinfectants or antiviral agents.

**Figure 2:**
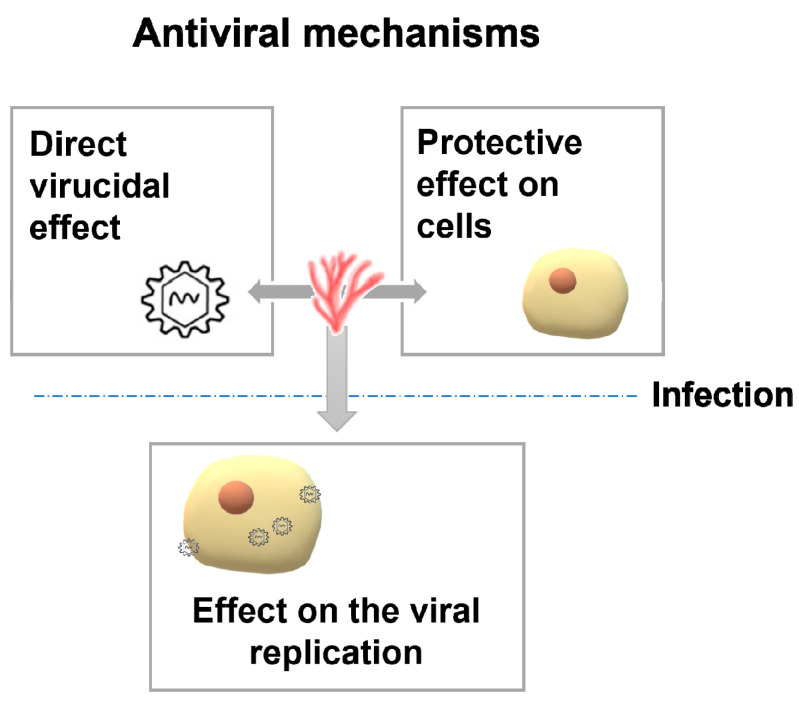
Antiviral effect of seaweed polysaccharides.

Carrageenans have been reported to be effective mainly against enveloped viruses. The most studied are members of the family *Herpesviridae* comprising herpes simplex virus types 1 and 2 (HSV-1 and 2) [23,39,44,62,63,64,65,66,67], cytomegalovirus (CMV) [63], varicela zoster virus (VZV) [14], equid herpesvirus 3 (EHV-3) [68], and bovine herpesvirus type 1 (BoHV-1) and suid herpesvirus type 1 (SuHV-1) [13]. Other enveloped viruses affected by carrageenans included dengue virus type 2 (DENV-2) [8,39,69], human immunodeficiency virus (HIV) [63,70], Sindbis virus [63], Influenza virus (IAV) [16], human metapneumovirus (HPNV) [11], porcine reproductive and respiratory syndrome virus (PRRSV) [25], rabies virus (RABV) [46], Rift Valley fever virus (RVFV) [67], vaccinia virus [62,63], Semliki forest virus and swine fever virus [62], hantaviruses [71], and the fish virus viral haemorrhagic septicaemia virus (VHSV) [unpublished results]. In addition, recent studies have demonstrated that iota and lambda carrageenans have a potent inhibitory activity against SARS-CoV2 [61,72,73], and other sulfated polysaccharides have been postulated as candidates for prevention and/or treatment of the COVID-19 [36,74,75]. Some authors have also reported activity against non-enveloped viruses such as human rhinovirus (HRV) [76], enterovirus 71 (EV-71) [24], and papillomavirus type 16 (HPV-16) [77]. An illustrative scheme of the antiviral action of seaweed polysaccharides is summarized in Figure 2, a compilation of antiviral activities shown by carrageenans is presented in Table 2, and more extensive information can be found in recent comprehensive reviews (e.g., [32]). However, the basis of this antiviral activity has not been clarified in many cases.

Antiviral mechanisms include a direct virucidal effect, an effect on the viral replication, and/or a preventive effect on the susceptible cells. Studies regarding each of these putative mechanisms are reviewed in this section.

### 3.1. Direct Virucidal Effect

Although it has been reported that carrageenan has a direct virucidal action against a variety of enveloped viruses [29], only a few research articles have truly tested the virucidal potential of sulfated polysaccharides (SPs) in general and carrageenans in particular. Most of these studies have been performed with herpes viruses, which were inactivated by different algal extracts.

Harden and co-authors reported virucidal activity against herpes simplex virus-2 (HSV-2) of carrageenans obtained from the red alga *Gigartina atropurpurea* and other SPs extracted from *Splachnidium rugosum* and *Plocamium cartilagineum* and to a lesser extent from *Undaria pinnatifida* [23]. The antiherpetic activity of λ-carrageenans extracted from other *Gigartina* species, *G. skottsbergii*, has also been demonstrated with two animal herpesviruses: bovine herpesvirus-1 (BoHV-1) and suid herpesvirus-1 (SuHV-1) [13]. In addition, inactivation of herpes simplex virus-1 (HSV- 1) and Rift valley fever virus (RVFV) was reported by Gomaa and Elshoubaky using carrageenans obtained from *Acanthophora specifira* and *Hydroclathrus cathralus* [67]. Finally, Abu-Galiyun and co-authors reported 85% inhibition of VZV infectivity when treated with ι-carrageenans [14].

### 3.2. Effect on the Viral Replication

**Figure 3:**
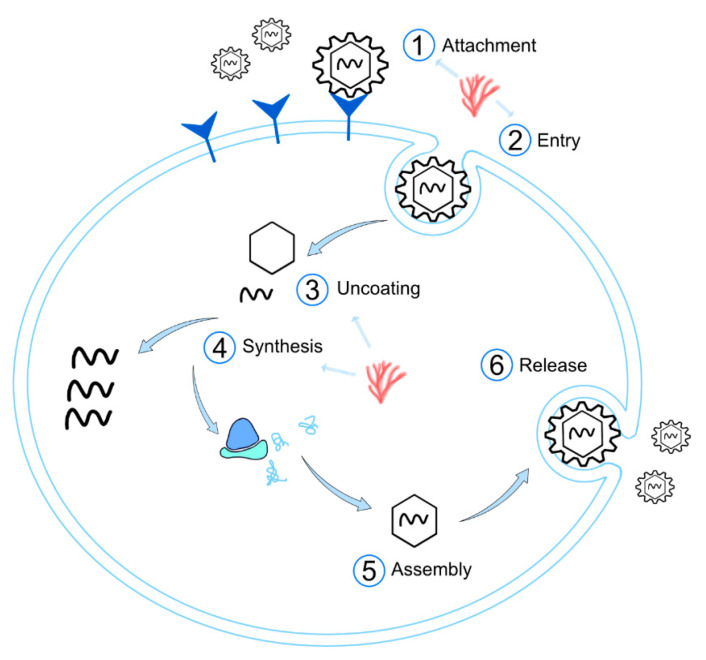
The process of viral infection and the antiviral effect of seaweed polysaccharides (adapted from [27]).

As it is shown in Figure 3, the viral cycle comprises six steps: (1) cell adsorption or attachment, (2) entry, (3) uncoating, (4) synthesis (replication and translation of viral genomes), (5) assembly, and (6) release. Most reports have studied the effect of carrageenans in the two first steps in different virus and cell lines, but an inhibitory activity against viral synthesis has also been described. A summary of the studies focused on the blocking of each of the steps of viral replication is given below.

#### 3.2.1. Inhibition of Viral Adsorption

Viral infection requires binding to the host cell surface. A variety of cell surface components can play a role in viral attachment, and among them, glycans displayed on the protein or lipid surface interact with a high number of viruses [83,84]. Glycans involved in viral binding comprise three families: histo-blood group antigens (HBGAs), sialoglycans, and glycosaminoglycans (GAGs). GAGs, which are long polysaccharides composed of sulfated uronic acid and glucosamine residues, are recognized by different enveloped viruses such as herpesviruses, Dengue, and Zika [84,85] and non-enveloped viruses such as HPV and human parvoviruses [84]. It has been shown that the interactions between GAGs, particularly heparan sulfate (HS), and different enveloped and non-enveloped viruses can be blocked by carrageenans, especially the λ-, ι-, and κ-forms [1,8,11,12,14,23,24,25,42,44,63,69,76,78]. In the enveloped viruses, this blocking capacity has been reported to be likely due to the carrageenans ability to bind the viral envelope’s proteins, such as herpes simplex glycoprotein or influenza hemaglutinin, preventing virus binding to GAGs [1,64]. A similar mechanism, which consists of blocking the interaction between the viral capsid and cell surface receptors, seems to be the explanation for the activity against non-enveloped viruses [76,77] due to the occlusion of virion surfaces involved in binding to cellular receptors.

#### 3.2.2. Inhibition of Viral Internalization/Entry

After virus attachment to the cell surface, viral proteins undergo conformational changes, which activate signaling cascades and destabilize the cell membrane, leading to viral internalization either by an endocytic or non-endocytic route [86,87].

Only a few studies have reported the effect of carrageenans on viral entry, probably because its demonstration requires the use of more sophisticated techniques. Talarico and Damonte quantifying viral RNA inside the cells demonstrated that λ-carrageenans can block DENV-2 nucleocapsid internalization into the cytoplasm [8]. More recently, Piccini and co-authors reported that these compounds are potent inhibitors of the internalization of the four DENV serotypes in human myeloid U937 and K562 cells, both in primary and antibody-dependent infections [88]. Similarly, λ-carrageenan P32 has been shown to affect RABV virus internalization [46].

HSV-1 internalization was reported to be blocked by another type of carrageenans, ι-carrageenans, using radiolabeled viral particles [62]. These compounds can also have an inhibitory post attachment effect against two non-enveloped viruses, HPV and HRV, either by the occlusion of virion surfaces involved in binding to cellular receptors or by hampering obligatory conformational changes in the viral particle [76,77].

#### 3.2.3. Inhibition of Uncoating

To the best of our knowledge, only two reports point to the antiviral effect of carrageenans in this step. Talarico and Damonte suggested that the blocking of DENV-2 internalization by λ-carrageenans is produced, likely because virions that enter the cells are not able to be uncoated and released from the endosomes [8]. Luo and co-authors reported that the λ-carrageenan P32 could inhibit the conformational changes of RABV glycoprotein, blocking cell fusion mediated by this protein and inhibiting viral uncoating [46].

#### 3.2.4. Inhibition of Synthesis

Initial reports in the 1980s indicated that carrageenans’ polysaccharides affected the protein synthesis and enzymatic activities of HSV-1 and avian mieloblastosis virus (AMV), respectively [45,62]. Thus, González and co-authors reported that viral protein synthesis was declined when HeLa cells were infected with HSV-1 in the presence of carrageenans, whereas cellular proteins were not affected [62]. Nakashima and co-authors demonstrated that a λ-carrageenan obtained from *Schizymenia pacifica* inhibited the reverse transcriptase activity of AMV [45].

More recently, the effect of carrageenans on intracellular replication steps has been thoroughly studied in influenza A virus (IAV) [1,81,82]. Low-molecular-weight (LMW) κ-carrageenans have been demonstrated to enter Madin Darby canine kidney (MDCK) [81] and to inhibit both IAV transcription and protein expression, probably by interfering with viral polymerase activity [1,81]. κ-carrageenans have also shown to be able to suppress EV 71 mRNA synthesis in Vero-infected cells [24].

Finally, the putative effect of ι-carrageenans in DENV and VZV intracellular replication steps has been suggested [14,69]. Abu-Galiyun and co-authors reported 75% inhibition of VZV intracellular replication in Vero cells, and Talarico and co-authors reported that ι-carrageenan could inhibit the replication of DENV in the C6/36 HT mosquito-derived cell line by affecting potential targets within the host cells [14,69].

### 3.3. Protective Effect on Cells

Carrageenans’ prophylactic activity has been seldom analyzed, probably because most of the studies indicated that these compounds exert an antiviral activity by binding the viruses and preventing them from adsorbing to cell cultures. Chiu and co-authors found that pre-treatment of Vero cells with κ-carrageenan for 1 h before infection with EV 72 showed a potent viral inhibitory effect (92%), slightly higher than that of the group treated at the infection time (87%) or post-infection (82%), suggesting that the carrageenan molecule can bind to both the cell receptor and viral surface [24]. Talarico and co-authors also observed that pre-treatment of C6/36HT mosquito cells with ι-carrageenan caused a reduction of 1.5 log in the virus yield at 48 post infection and that the EC_50_ value was similar to that obtained when the virus and carrageenan were incubated for the whole infection period [69]. Pavliga and co-authors observed a reduction in the titer of hantavirus PM-95 of 1.8, 1.4, and 1.4 log focus forming units (ffu)/mL, in Vero E6 cells pretreated with κ-, λ-, and ι-carrageenans during 1 h [71]. On the contrary, Vero cells were not protected from HSV-1 or VZV infection when the polysaccharides were present only before virus infection [14,20].

## 4. Determination of the Antiviral Activity

Different approaches to assessing the antiviral activity of carrageenans have been described in the literature, although all share the need to rule out the cytotoxicity of the compound/concentration used and quantify the virus before and after treatment (viral yield assay). Cytotoxicity is mainly determined by colorimetric methods such as 3′-[4,5-dimethylthiazol-2-yl]-2,5-diphenyltetrazolium bromide (MTT) and neutral red and crystal violet staining, and results are expressed as the cytotoxic concentration 50% (CC_50_), the compound concentration, which reduces cell viability to 50%. Viral quantification can be accomplished by plaque-forming units (PFUs) [8,13,14,23,24,38,39,42,44,62,64,65,67,68,78,88,89,90,91] or by the Tissue Culture Infection Dose 50 method (TCID_50_) [25,46,76,81]. However, other procedures have been used to determine and characterize the antiviral activity including colorimetric methods [1,20,63,66,76,81], ELISA [24], flow cytometric analysis [11,77,78], haemagglutination inhibition assay [1,28], immunofluorescence assay [46,69,92], RT-PCR [8,25,67,93], and Western blot [25,46].

**Figure 4:**
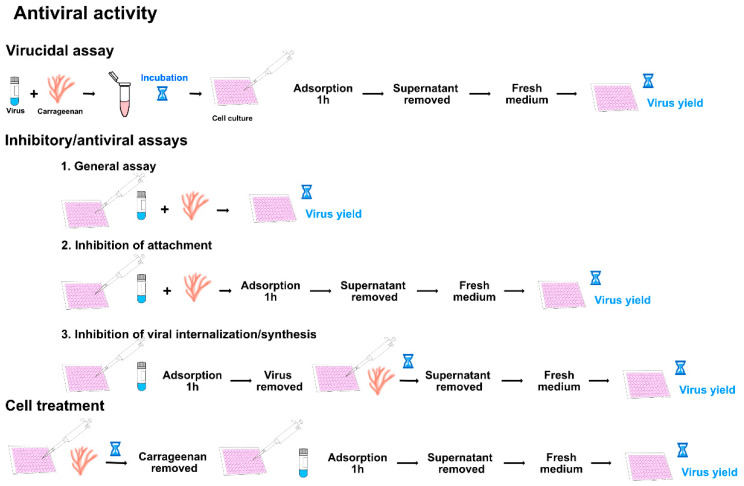
Simplified scheme of the antiviral activity assays.

Regarding antiviral activity assays, the procedures are different depending on whether virucidal or antiviral activity is to be determined. Carrageenan’s virucidal activity has been scarcely reported [11,13], and it is studied by incubating virus and carrageenan for a certain time followed by quantification of the virus in cell culture (Figure 4). The result, compared to the quantification of the untreated virus, can be expressed as virucidal concentration 50 (VC_50_) defined as the concentration required to inactivate virion to 50%. Most of the published studies have analyzed the antiviral activity of carrageenans either in a general way, without identifying the affected step of the viral cycle, or as a previous step to a further characterization [8,11,23,25,38,44,66,69,76,78] (Figure 4, step 1). In these assays, cell monolayers are infected in the presence/absence of carrageenans, and after different incubation times, virus yield is determined. Several studies have assessed the inhibition of viral attachment to the susceptible cells [1,8,11,12,13,14,23,24,25,39,42,44,63,69,78,94]. To this purpose, cell cultures are infected with the virus in the presence/absence of the carrageenan and after 1 h adsorption the inoculum is removed, and new media are added (Figure 4, step 2). In those studies, which analyzed the blocking of viral entry or further replication steps, viruses and carrageenans were incubated for longer periods of time [1,8,14,24,69,76,81,82,88,93] (Figure 4, step 3). A few studies analyzed the effect of carrageenan addition to cell cultures by incubating cells with the carrageenan and removing it before viral infection (Figure 4).

In all antiviral assays, results are expressed as the inhibitory concentration 50% (IC_50_) or, more frequently, as the effective concentration 50% (EC_50_), both defined as the compound concentration required to reduce virus yield by 50%. In addition, the selectivity index (SI), a ratio that measures the relation between cytotoxicity and antiviral activity (CC_50_/EC_50_), is a widely accepted parameter to express the efficacy of an antiviral compound. The higher the SI value, the more effective and safer the agent should be. Although SI ratios are not frequently used to measure the antiviral activity of carrageenans, reported values include SI ranging from to 28 to <200 (κ-carrageenan oligosaccharides and of p-KG03, respectively) when tested against IAV [28,95] and 62.9 in the case of the activity of the λ-carrageenan P32 against RABV.

## 5. In Vivo Studies and Medical Applications

### 5.1. Animal Models

The antiviral activity of carrageenans has also been analyzed in different animal models with very satisfactory results, mostly murine and *Macaccus rhesus*. Experiments using the murine model have demonstrated the antiviral effect of λ and κ-carrageenan against IAV infection [28,78]. Intranasal treatment with λ-carrageenans caused an immediate reduction of IAV particles in the nasal cavity after infection and improved mice survival [78]. In a similar way, Wang and co-authors reported that κ-carrageenan oligosaccharides improved survival and decreased pulmonary viral titers in the infected animals [82]. This model has been also used to demonstrate λ-carrageenan and zinc carrageenan gels protection against disease caused by intravaginal HSV-2 challenge [44,80,96,97]. Finally, the effectivity of carrageenans to prevent genital HPV infections has also been demonstrated [98]. The experiments with *Macaccus rhesus* have included the use of other compounds and are detailed in Section 5.3.

### 5.2. Medical Applications

In recent years, several clinical trials designed to test the antiviral efficacy of carrageenans against different viral infections have been reported with different results [99,100,101,102,103]. A phase III clinical trial was performed to assess the efficacy for preventing HIV infection of Carraguard (PC-515), a gel made from a carrageenan (product number PDR98-15 FMC, Philadelphia, PA, USA) in South Africa [101]. This trial, with 6202 participants, did not show Carraguard’s efficacy in the prevention of vaginal transmission of HIV. However, in another trial, Marais and co-authors suggested that Carraguard could be used to prevent HPV infections [99]. In different studies targeted to viral respiratory viruses causing the common cold, a ι-carrageenan spray showed a good effectivity in reducing the severity of the disease symptoms in adults [100,101,104]. In these studies, patients were demonstrated to be infected by HRV, human coronavirus (HCoV), or IAV [101], the previously cited virus and human parainfluenza virus (HPIV) [104] and respiratory sincitial virus (RSV), HCoV, human metapneumovirus (HMPV), HRV, and HPIV [100]. Carrageenan nasal spray showed significant antiviral efficacy against all detected common-cold viruses [100,101,104] but especially against HCoV [101], which was evidenced by the reduced duration of disease, increased viral clearance, and reduced relapses compared with placebo-treated patients [101]. A similar study performed with children revealed that although ι-carrageenan nasal spray did not alleviate symptoms, it significantly reduced viral load in nasal secretions [102]. Recently, nasal spray formulations containing λ- or ι- and κ-carrageenans and an oral spray containing ι-carrageenan have been demonstrated to be useful for preventing SARS-CoV-2 infection in Vero cells and epithelial airway cultures [105,106].

Finally, it is interesting to notice that non-medical applications have also been reported, i.e., for film-forming dispersions (FFD) of κ- ι- and λ-carrageenans for coating blueberries and raspberries stored refrigerated and at room temperature. The FFD not only acted as an antiviral against murine norovirus (MNV) and hepatitis A virus (HAV) but also extended the shelf life, maintained firmness, and improved the berries’ appearance. The type of carrageenan used significantly affected the mechanical properties of the films, k-carrageenan films being more rigid and less permeable, providing higher antiviral activity [107]. Additionally, agricultural applications have been proposed, based on the antiviral activity against tobacco mosaic virus [108].

### 5.3. Combination with Conventional Drugs

The problem of antiviral resistance could be addressed with a strategy that combines antivirals targeting different steps of viral replication [109]. Leibbrandt and co-authors reported the additive therapeutic effects of iota-carrageenan and Oseltamivir, a viral neuraminidase inhibitor to synergistically promote survival of mice using an intranasal application 48 pi [78]. Combination treatments showed significantly increased survival rates as compared to a monotherapy with either iota-carrageenan or Oseltamivir. Synergistic interaction of carrageenan and other neuraminidase inhibitor, Zanamivir, against IAV, H1N1, and H7N7 infections has also been reported [91]. The combined therapies provided higher activity against IAV strains than the individual compounds and led to 50–90% survival, depending on the Zanamivir concentration.

The antiviral activity of carrageenans in combination with different compounds has been tested against sexually transmitted viruses. Levendosky and co-authors reported that the use of carrageenans combined with Griffithsin (GRFT), a lectin with a high affinity for mannose-rich N-linked glycans, shows a broad antiviral activity against HSV-2 and HPV infection in mice [110]. An intravaginal ring (MZC), which releases carrageenan, zinc acetate, and the antiviral agent MIV-150, protects macaques against vaginal challenge with simian immunodeficiency virus (SIV) containing the reverse transcriptase gene from HIV (SHIV-RT) [111]. Subsequent studies demonstrated that MZC significantly prevented SHIV-RT infection and reduced HSV-2 vaginal shedding [112]. In addition, the combination of carrageenan and GRFT was tested by using a freeze-dried fast-dissolving insert (FDI) formulation and was demonstrated to protect rhesus macaques from a high-dose SHIV vaginal challenge [113].

Other combination strategies have been focused on in reducing clinical signs. This was the approach followed by Graf and co-authors who reported that ι-carrageenan retains its antiviral effectiveness against respiratory viruses in the presence of xylometazoline HCl and does not affect the vasoconstrictive efficacy or the permeation behavior of xylometazoline HCl [114]. The combined formulation (0.05% X/0.12% ι-C) in a nasal spray was well-tolerated upon repeated intranasal administration and showed both vasoconstrictive properties and antiviral effectiveness against HRV and HCV infections.

Synergistic antiviral action was also reported for green tea extracts and carrageenan used as edible coatings to protect fruits during storage and to lower the viral titer of MNV and hepatitis A virus (HAV) [107].

### 5.4. Patents Claiming the Use of Carrageenan

Carrageenans can be included in pharmaceutical compositions for the prevention or treatment of viral infections with no side effects [115,116]. Some examples could be mentioned. Ungheri and co-authors claimed a composition comprising a fibroblast growth factor, a sulfated polysaccharide with antiviral activity, such as carrageenan, and one or more pharmaceutically acceptable carriers [117]. Enomoto and co-authors proposed a condom with prophylactic action against AIDS infection coated with acidic sulfated polysaccharides, among them carrageenan [118]. Grassauer and co-authors used ι-, κ-, or λ-carrageenan or their mixtures for the manufacture of a pharmaceutical composition for the prophylaxis or treatment of a rhinovirus infection, ι-carrageenan being the most efficient to reduce the risk of infection [119,120]. The use of ι- and/or κ-carrageenan has been included in antiviral compositions for the prophylaxis or treatment of infection by respiratory virus (orthomyxovirus, paramyxovirus, adenovirus, and coronavirus) [119]. Grassauer and co-authors have claimed the use of carrageenans for deblocking a stuffy nose and for prophylactic or therapeutic intervention into viral infections of the upper respiratory tract [120]. Additionally, Bodenteich and co-authors proposed ι-carrageenan as an active antiviral ingredient in a pharmaceutical composition for prophylactic or therapeutic topical treatment of viral eye infections caused by adenovirus of subtype D or IAV of subtype H7N [121]. Lambda-carrageenan was used as anti-viral agent, for protection from HPV, in lubricating compositions [122], and ι-carrageenan was the active antiviral agent against herpes virus in a pharmaceutical composition for external application [123].

Park and co-authors designed a composition for preventing or treating rhinovirus or IAV infectious diseases and a functional food composition for alleviating or mitigating the diseases [115]. Vestweber and co-authors developed a solid composition comprising 0.1–40 wt%, preferably 3–18 wt% virustatic and/or antiviral polysaccharide [124]. Carrageenan has also been proposed to bind a light-activated non-leachable dye, which upon exposure to normal light generates singlet oxygen that inactivates viruses [125].

Carrageenan-derived oligosaccharides have been incorporated with other plant and marine bioactive compounds in antimicrobial and antiviral oral liquid for regulating metabolism and enhancing immunity [126]. Deng and co-authors designed a sugar-free healthcare candy with antibacterial and antiviral effects, prepared with plant extracts, fruits, sweeteners and gelatin, carrageenan, and pectin [127].

Additionally, the combination with conventional drugs was proposed; Grassauer and co-authors reported the combination of ι- and/or κ-carrageenan with a neuraminidase inhibitor (zanamivir, oseltamivir, peramivir and laninamivir) for the treatment of symptoms and conditions associated with an infection by IAV [128].

## 6. Factors Influencing Antiviral Properties

In order to adequately select the most active carrageenan type, different aspects have to be considered: (i) the virus type and the characteristics of the capsid/envelope proteins, (ii) the host cell type, and (iii) the carrageenan composition and structure, which depends on the seaweed species and can also be altered during processing [28,42,64,114]. In relation to the composition and structural aspects of carrageenan, particularly relevant are the (i) type of carrageenan, particularly the content of α-d-galactose 2,6-disulfate residues, being highest in λ- and ν-carrageenans, and the molar ratio of galactose to 3,6-anhydrogalactose, highest in lambda- and μ/ν-carrageenans; (ii) the sulfation degree and the position of the sulfate group, which is on C-2 in lambda-carrageenans and on C-4 in κ/ι- and μ/ν -carrageenans; and (iii) the molecular weight [28].

The most active oligomers have a definite range of sulfate contents and molecular weights regardless of the particular type of sulfated carrageenan [9,23,129,130,131,132]. The influence of virus strain and host cell type have been recently reviewed, and in this section, the importance of carrageenan composition and structure in relation to processing the antiviral activity will be discussed.

### 6.1. Type of Carrageenan

The antiviral effect of carrageenans depends on their structures, which also define their gelling type. Lambda is usually the most active; it has been suggested that it is related to the sulfate content [40]. When Nakashima and co-authors compared the efficiency of carrageenans as reverse transcriptase inhibitors of HIV and replication in vitro, they observed the decreasing order λ-carrageenan, ι-carrageenan, and κ-carrageenan [45]. The λ-carrageenan is a potent and selective inhibitor of virus infection targeted to virus adsorption and internalization, due to the structural similarities with the mammalian cell receptor heparan sulfate [79]. However, no effect of the addition on latter stages of infection was found [8]. Abu-Galiyun and co-authors also reported a decreasing order of activity for λ, ἰ, G, κ, and P against VZV-resistant strains [14]. A comparable effect has also been reported for oligomeric fractions. Carlucci and co-authors reported that ἰ -carrageenan added after 1 h of infection still produced a significant reduction in HSV-1 multiplication in Vero cells, whereas the κ:ι-carrageenan 1C1 and the µ:υ-type were ineffective when added after virus attachment [42]. Different carrageenan types can have a different inhibition mechanism, and the activity can be different depending on the moment of addition. Synergistic action between different carrageenan types was confirmed with the better therapeutic effect resulting from the combination of iota- and κ-carrageenan in comparison to the use of iota-C alone for the treatment of influenza in C57Bl/6 mice [91].

### 6.2. Sulfate Content

Sulfate content in carrageenans varies from 25–30% in κ-carrageenan to 28–30% in ι-carrageenan and 32–39% in λ-carrageenan [40]. Among the three sulfated carrageenans, the sulfate content of lambda-carrageenan is the highest. Sulfated polysaccharides with sulfate content higher than 20% tend to show higher antiviral activity. More sulfated κ-carrageenan and its LMW derivatives had higher antiviral activity than κ/β-carrageenan and its oligosaccharides [108]. However, the existence of an optimal sulfation degree could be inferred from the fact that the sulfation of κ-and ι-carrageenan increased their activity to values comparable to that of λ-carrageenan, but no increase was noticed after sulfation of λ-carrageenan, and the most active preparations had a sulfate content of 3.4–3.8 mole/mole of disaccharide [132]. The influence of this variable is also evidenced by the fact that desulfation of the original carrageenans lowered their anti-HIV activity [70].

Yamada and co-authors found a different anti-HIV potency for λ-carrageenan and ι-carrageenan, even when their sulfate content was similar, suggesting that the structural features were also highly influencing on the anti-HIV activity [132]. The number of sulfate groups as well as the molecular weight of the biopolymer play an important role in the anti-HIV activity of the sulfated carrageenans, suggesting that the activities were not entirely dependent on sulfate content; the positions and densities of the sulfate groups on the sugar chains can be important factors [70].

### 6.3. Molecular Weight

The high-molecular-weight carrageenan has good inhibitory effects on virus replication, particularly acting on the initial attachment stages [1]. However, these sulfated polysaccharides have poor tissue-penetrating ability, making it improbable to cross the cell membranes. The external or local applications are more promising [78]. Lower molecular weight oligosaccharides display low viscosity and high solubility at neutral pH, and their ability to enter cells could provide enhanced antiviral properties [70,82,132].

The different action modes between carrageenan polysaccharides and oligosaccharides might be attributed to the molecular structures and their abilities to enter cells [28]. In addition to blocking the adsorption and internalization, low-molecular-weight κ-carrageenan also inhibits mRNA transcription and protein expression due to the interference of polymerase activity [1]. A different mechanism of oligomeric fractions was suggested, since the 2 kDa compound did not bind to the cell surface of MDCK cells but inactivated virus particles after pretreatment, probably because this oligomer could enter cells and did not interfere with the adsorption step. Wang and co-authors also observed that the 2 kDa oligomer inhibited IAV mRNA and protein expression after its internalization into cells, and this effect on IAV replication occurred subsequently to viral internalization but prior to virus release in one replication cycle, inhibiting virus life-cycle steps that occur 0–4 h after infection [81].

Usually, the antiviral activity of polysaccharides correlates with their molecular weight [129,130]. Optimal MW has been reported for 1–4 kDa oligomers. Luo and co-authors selected different λ-carrageenan saccharides (4–330 kDa), which significantly inhibit the infection of the RABV strainSAD-L16-eGFP, and found the highest effect for a low-molecular-weight (4 kDa) λ-carrageenan oligosaccharide (CO) with high solubility and high stability [46]. It has been suggested that polymers of a certain length might be essential for the antiviral activity of κ-carrageenan oligosaccharide [28]. Later authors reported higher inhibition of IAV (H1N1) virus replication in MDCK cells by a 2 kDa κ-carrageenan oligosaccharide than by those with higher MW (3 and 5 kDa), and the selectivity index was similar to that of ribavirin. They possessed mainly 1,4-linked β-d-galactose and 1,3-linked α-d-galactose with about 0.4 sulfate per sugar residue. Wang and co-authors found better protection from virus-induced cell death in cells treated with 2 kDa κ-CO than in those treated with 4 kDa κ-CO, and the inhibition percentage of 1 kDa κ-CO was even lower [82]. The selectivity index for 1 and 4 kDa was no more than 3.0, whereas the 2 kDa showed a SI of 27. They also confirmed that the degree of sulfation and molecular weight were the main factors affecting the anti-IAV activity in MDCK cells of κ-carrageenan oligosaccharide, the optimal sulfate content of 0.8–1.0 mole/mole of disaccharide, and a molecular weight of 1–3 kDa. Wang and co-workers found that carrageenan oligosaccharide made from carrageenan by chemical or enzymatic degradation has smaller molecular weight and is easy to contact with viruses; thus, its bioavailability and biological activity is significantly increased [28]. Tang and co-authors found that low-molecular-weight carrageenans and their derivatives showed significant inhibition effects against IAV FM1-induced pulmonary edema in mice, and the 3 kDa k-carrageenan with proper acetylation degree and sulfation degree possesses best antiviral activity in vivo [133].

## 7. Processing

### 7.1. Extraction

After a preliminary stage to remove impurities, the extraction of red seaweed polysaccharides is usually performed on dried algae. Carrageenan extraction is performed in a hot aqueous solution, under neutral or alkaline conditions. The resulting suspension is filtered, and the carrageenan can be recovered from the solution by alcohol precipitation and further dried and milled. Optimization of the major variables (temperature, pH, time, alkaline agent type and concentration, and time) is needed in order to maximize the structural and gelling properties. Intensification technologies have been proposed to enhance the efficiency and to lower time, energy demand, and consumption of chemicals. Among them, pressurized solvent extraction, microwave-, ultrasonic- and enzyme-assisted extractions have been tried [134,135].

Compared to the conventional method, the use of microwave-assisted extraction allows similar yields in shorter times and with lower alkali concentration during pretreatment [136] and provides increased antiviral activity of *Soliera chordalis* ι-carrageenan against HSV-1 [66]. No changes in the chemical structure due to microwave radiation were evident compared to carrageenan extracted under the conventional method; the total sulfate and d-anhydrogalactose (D-AHG) contents in carrageenan treated with 1% KOH under different microwave conditions were close to the values for commercial ι-carrageenan. The maximum anti HSV-1 activity was displayed by a carrageenan with high sulfate content and a low proportion of D-AHG.

The ultrasound assistance lowered the extraction times compared to the conventional method without affecting the chemical composition and structure. Ultrasound positively contributed to depolymerization of κ-carrageenan involving an enhancement in the corresponding biological activities [137]. A sonication operation at a wide range of pH values (3–6), temperatures (30–60 °C), and times (<24 min) promoted an adequate depolymerization of carrageenans, obtaining oligocarrageenans with low molecular weights, which are relevant for biomedical applications [138]. The depolymerization of this kind of biopolymers using ultrasound-assisted extraction in acid media also increased the anticoagulant activity [139].

### 7.2. Depolymerization

Despite having excellent biological activities, the high-molecular-weight polysaccharides possess high viscosity but poor tissue penetrating ability and bioavailability, which limit their therapeutic potential [108]. In order to improve both their bioavailability and biological activity, carrageenans have been degraded into oligosaccharides with lower molecular weight [140]. LMW carrageenan derivatives with different MWs can be obtained by mild acid hydrolysis, free radical depolymerization, or enzymatic degradation [133,135]. The LMW derivatives prepared by different methods have various chemical structures as a result of different mechanisms of the different depolymerization processes [108], which also influences their biological profile [40,69,132]. Carrageenan oligosaccharides exhibit promising biological properties and potential pharmacological and therapeutic applications [10,33,38,39,40,44,64,69]. The conditions for depolymerization of carrageenan aided by acid, enzymes, high pressure, microwave, ultrasound, ozone, or irradiation and the range of molecular weights of the resulting carrageenan oligomers can be found in some reviews [135,138,139].

#### 7.2.1. Mild Acid Hydrolysis

Dilute acids, such as hydrochloric acid, nitric acid, sulfuric acid, trifluoroacetic acid (TFA), formic acid, tartaric acid, and lactic acid, after being heated to 50–90 °C, can be employed for the production of oligosaccharides from carrageenan. The chemical degradation only led to the cleavage of the glycosidic bond and did not destroy the disaccharide structure. A random cleavage of glycosidic linkage was observed after depolymerization with an acid solution of TFA at 65 °C for 3 h [33]. The mechanism of mild acid hydrolysis implies breaking inside α-1.3 links to produce odd-numbered oligosaccharides (-G4S-(AHG-G4S)n-) and breaking of β-1.4-linked oligosaccharides to yield even-numbered fragments (-(G4S-AHG)n-) in minor amounts [33,141].

Mild acid hydrolysis yielded fragments with molecular weight from 1.2 to 3.0 kDa. The increase in temperature for mild acid hydrolysis up to 60 °C promoted the decrease in sulfate groups and 3.6-AnGal contents due to a partial destruction under high temperature [108].

Excessive depolymerization may lower the antiviral potency. The oligosaccharide fractions of κ/β-carrageenan (1.2, 1.7, and 3.0 kDa) obtained by acid hydrolysis under various conditions showed similar activity, but only polymers with high molecular weight (250, 390, and 400 kDa) inhibited local necrosis in tobacco leaves. κ-carrageenan oligosaccharides of 1 kDa were less efficient to lower the cytopathic effect of IAV PR/8 strain and to promote the survival of IAV-infected MDCK cells than the 2 kDa oligosaccharide [82]. Probably, the treatment caused undesirable modifications in other features. During mild-acid hydrolysis with 0.1 M HCl in the presence of LiI or LiCl, for κ- and ι-carrageenan, respectively, significant desulfation was found to occur. Furthermore, the rate constant for the hydrolysis was found to differ for both the carrageenan type and the nature of the ordered and disordered conformation [142]. Cleavages of glycosidic linkages in multiple-stranded polymers are different than in single-stranded polymers, which suffer a more marked decrease in the molecular weight. However, after a 10-fold decrease in molecular weight, extensive disintegration of the multiple-stranded structure occurs, and the decrease in molecular weight is faster than for single-stranded chains, even if the rate of bond cleavage is the same [143].

The antiviral activity can be modulated with conditions during processing and also by further refining. Laporte and co-authors prepared oligosaccharides from red algae through chemical depolymerization, differentially stimulated growth of plants, and defense against tobacco mosaic virus in these plants [144]. Partial depolymerization of a d-, l- hybrid galactan (agaran:carrageenan present in a molar ratio of 1.7:1) using borane-4-methylmorpholine complex at a final concentration of 0.375 M TFA at 65 °C and further chromatographic fractionation enhanced the activity against HMPV (human metapneumovirus) [145].

Chemical hydrolysis is simple, fast, reproducible, and scalable, but has disadvantages derived from the low specificity, the production of high amounts of monosaccharides, and the generation of undesirable brown products and toxic compounds, as well as the associated environmental hazards [33].

Microwave heating was proposed to enhance the acid depolymerization of native κ-carrageenan and to shorten the reaction time. The average MW of LMW κ-carrageenans decreased with increasing microwave heating intensity and decreasing pH value, but operation in open systems did not cause desulfation or change in the structure and constituents. The MW of native κ-carrageenan, 4000–5000 kDa, was lowered under 50 kDa with a narrow molecular weight distribution. Even narrower distributions of LMW κ-carrageenans (3–10 kDa) could be obtained under pH 2.18 using a domestic microwave oven. The products showed significant inhibition against IAV-induced pulmonary edema, although their activities were inferior to that of Ribavirin [133]. The λ-carrageenan polysaccharides prepared by microwave degradation could significantly improve the activity of NK cells and enhance the proliferation rate of lymphocytes [146]. Closed operation can be even more rapid, but desulfation may occur [147]. Antiviral activity of *S. chordalis* carrageenan, extracted using microwave treatment, against HSV-1 promoted lower EC_50_ of the ι-carrageenans fractions (3.2–54.4 μg/mL) without cytotoxicity in that range of concentrations [66]. Combination with membrane fractionation can be useful to obtain a desired range of MW, i.e., 400 Da–50 kDa κ-carrageenans can be readily prepared by microwave-aided acid hydrolysis followed by nanofiltration [133].

#### 7.2.2. Subcritical Water Extraction

Water processing at temperatures in the range from 120 to 374 °C is normally referred to as subcritical water, which is considered a green extraction technology, allowing simultaneous extraction and depolymerization. Different operation conditions have been proposed in the literature. Gereniu and co-authors reported that the κ-carrageenan extracted from *Kappaphycus alvarezii* by SWE using ionic liquids as catalyst, provided higher yields due to the enhanced depolymerization [148]. However, the sulfate content and the gel strength of the biopolymer were lowered compared to those of the conventional method [148]. Bouanati and co-authors accelerated the hydrolysis (5 min) of carrageenans from *Kappaphycus alvarezii* and *Eucheuma spinosum* in pure water using microwave activation, preserving the active sulfate groups [149]. In this context, *M. stellatus* was processed from 70 to 190 °C for 3 and 6 min to extract hybrid carrageenan and antioxidants. The maximum value for antioxidant compounds was recovered at 190 °C, whereas the highest yield of carrageenan was obtained at 150 °C for 6 min; on the other hand, the strongest hydrogels were obtained at 170 °C for 6 min [56].

#### 7.2.3. Enzymatic Depolymerization

Carrageenases are enzymes produced by marine bacteria and degrade carrageenans, acting as endohydrolases, by hydrolyzing the internal β 1,4 linkages, producing a series of homologous even-numbered oligosaccharides [40]. They are classified into κ-, ι-, and λ-carrageenases. κ-Carrageenases act by a mechanism involving carboxylic acid-containing amino acid residues, promoting a double-displacement reaction and yield a series of homologous even-numbered oligosaccharides. ι-Carrageenases produce a series of homologous, even-numbered oligosaccharides, and λ-carrageenases lead to higher amounts of DP6s (or higher) compared to κ- and ι-carrageenases [40]. Other enzymes, such as β-galactosidases and cellulases, have also been used to produce oligosaccharides from red seaweed polysaccharides.

Marine bacteria have a large number of sulfatase enzymes, which play an important role in the degradation of algal sulfated polysaccharides. Biodegradation of carrageenans involves carrageenases, which cleave the glycosidic bond, and sulfatases, which catalyze the removal of sulfate groups according to either a hydrolytic mechanism or an oxidative mechanism [40]. Sulfatases can act after enzymatic depolymerization of carrageenan, specifically hydrolyzing sulfate ester groups located at the non-reducing end of oligo-carrageenans, but can also show an endo action to randomly remove the sulfate groups along the polysaccharide chain. Endo-acting carrageenan sulfatases act preferentially toward polysaccharides over oligosaccharides. Ghanbarzadeh and co-authors compiled a summary of important features and purification of different types of carrageenases from various marine bacteria [40]. Most of them showed maximal activity in the temperature range from 30 to 40 °C and optimum pH from 5.6 to 8.3. Most carrageenases’ activity loss is larger at temperature higher than 40 °C, when pH is more than 6.0. Some carrageenases present good thermostability, as is the case of λ-carrageenase from *Bacillus sp.* Lc50-1, which presents optimal activity at 75 °C. In recent years, some genes encoding these enzymes have been cloned, and the recombinant forms of different carrageenases were expressed in host cells and purified in large quantities and stable forms.

There has been a growing interest in converting carrageenans to their oligomers by carrageenases. Kalitnik and co-authors used a recombinant κ-carrageenase from *Pseudoalteromonas carrageenovora* over-expressed in *Escherichia coli* BL21 (DE3) and purified by affinity chromatography for the production of 2.2 kDa oligosaccharides from κ-carrageenan from *Chondrus armatus* and from *Kappaphycus alvarezii* and 4.3 kDa oligosaccharides from κ/β-carrageenan from *Tichocarpus crinitus* [108]. *Pseudomonas carrageenovora* was used to produce κ- and λ-carrageenases, and *Alteromonas fortis* was employed for ι-carrageenase [150].

A number of patented methods use this technology. Haijin and co-authors claimed a method for preparing sulfated oligosaccharide with activity against herpes simplex virus, obtained by hydrolysis with a crude digestive kappa-carrageenan and further sulfation [151]. Shao and co-authors claimed the preparation of an iota-carrageenan recombinase CgiA2_Ce applied to iota-carrageenan degradation to obtain two iota-carrageenan oligosaccharides (DP 2–4 and 6–10) with antiviral action, among others (antibacterial, immunomodulation, and antioxidant) [1]. Hong and co-authors claimed the production by a recombinant bacterial strain of a κ-carrageenase used to obtain oligosaccharides with antiseptic, antivirotic, immunomodulatory, and antioxidant properties [152].

Enzymatic depolymerization is advantageous, since the resulting oligosaccharides show higher homogeneity and lower polydispersity than other chemical methods, thus providing compounds with improved and reproducible biological properties [153]. The use of specific enzymes instead of radiation-induced or acid-promoted depolymerization avoids side reactions that can lead to undesired modifications of the native structure [33]. Enzymatic hydrolysis offers many advantages, such as specific hydrolysis resulting in the production of high amounts of target oligosaccharides, is environmentally friendly, and produces low amounts of monosaccharides and toxic molecules. However, it requires a long reaction time. Other disadvantages include the spontaneous loss of enzymatic activity and the high price, which suggest the need for enzyme reuse [33]. The results by Nakashima and co-authors illustrate the higher stability of a 2000 kDa λ-carrageenan from *Schizymenia pacifica*, composed of galactose (73%), sulfonate (20%), and 3,6-anhydrogalactose (0.65%). This compound was a selective inhibitor of human immunodeficiency virus (HIV) reverse transcriptase and replication in vitro when submitted to enzymatic hydrolysis with pronase, but the activity was reduced after boiling at 100 °C in the presence of 0.6 N HCl [45].

#### 7.2.4. Oxidative–Reductive Depolymerization

A well-known system for generating active oxygen species, used for the fragmentation of polysaccharides, consists of the combination of metal ions and ascorbate and is very effective to keep the ferrous ions in a reducted state. Yamada and co-authors proposed the depolymerization of λ-, κ-, and ι-carrageenan in the presence of ferrous ions or ferrous ions plus ascorbic acid at room temperature [132]. This strategy avoided the presence of trace amounts of contaminating Fe(II)/Fe(III) ions in final solutions. The anti-HIV activities of λ-carrageenan in MT-4 cells infected with HTLV-IIIB was similar to that of dextran sulfate, and the depolymerized ι-carrageenan retained high activity, whereas the activities of κ-and ι-carrageenan and their oligomers were much lower; the most active were those of 51 and 54 kDa, respectively. A small decrease in sulfate content was observed when samples were treated twice with ferrous ions and ascorbate, but the levels of anhydro galactose did not change during the depolymerization stage [132]. In a further study, Yamada and co-authors proposed the ultrasound-assisted depolymerization of λ- and κ-carrageenan at room temperature in the presence of ferrous ions, ascorbic acid, and ethylenediaminetetraacetic acid and observed higher anti-HIV action due to the increased antiviral activity with low anticoagulant properties and no hemolytic action [70].

#### 7.2.5. Hydrogen Peroxide (H_2_O_2_) Oxidation

The free radical depolymerization of carrageenan leads to a nonselective degradation producing a mixture of oligosaccharides with heterogeneous chemical structures and different molecular weights [108]. Hydrogen peroxide has been used for depolymerization of red seaweed polysaccharides, the structures of oligosaccharides obtained being usually -(G4S-AHG)n-, -AHG-(G4S-AHG)n-, -(G4S-AHG-OOH)n-, and -AHG-(G4S-AHG-OOH)n- [33]. During the preparation of low-molecular-weight polysaccharides from *Grateloupia filicina*, the hydrogen peroxide (H_2_O_2_) oxidation method, the degradation rate was favored by the H_2_O_2_ concentration and temperature and was negatively affected by pH. No change in the monosaccharide contents occurred; sugar and sulfate content decreased slightly. The depolymerized fractions, particularly that with 8.7 kDa, showed enhanced inhibition of avian leucosis virus ALV-J, mainly by blocking virus adsorption to host cells [92].

However, excessive depolymerization could occur; κ-oligosaccharides with molecular weight of 3.5 kDa obtained by free radical depolymerization had 1.3–1.5 fold lower activity than those from acid hydrolysis [108]. However, the H_2_O_2_ hydrolysates exhibited much higher antioxidant activities than the HCl hydrolysates [33].

#### 7.2.6. Autohydrolysis

This technology usually refers to a hydrolytic stage performed without the addition of chemicals at mild temperature conditions for prolonged times. Saluri and co-authors proposed the alkali treatment of native λ-carrageenan to obtain θ-carrageenan; autohydrolysis was performed for 0.5–72 h [154]. The molecular weight of λ-carrageenan samples ranged from 3100 to 4.7 kDa, whereas those of θ-carrageenan ranged from 630 to 1.1 kDa. The antioxidant activity increased with the decrease in molecular weight, and it was also influenced by the degree of sulfation and the presence of anhydrogalactose [154].

#### 7.2.7. Gamma Irradiation

Depolymerization of carrageenans can be achieved by irradiation with gamma rays at room temperature [140,155]. Relleve and co-authors reported that the susceptibility to degradation by irradiation with gamma rays at ambient temperature was influenced by the conformation being higher for λ, followed by ι and κ [156]. The molecular weights obtained were in the range of 8–105 kDa and the radiation-induced desulfation. Abad and co-authors found similar susceptibility of aqueous carrageenan to radiation degradation for κ-, ι-, and λ-carrageenan [140]. However, radiation-induced desulfation increased the acidity of the carrageenans, and the number of reducing end groups increased with dose. Greater susceptibility was observed in κ-carrageenan. The UV–vis spectrum of irradiated carrageenan showed a new peak at 260 nm, attributed to the formation of a carbonyl group or double bond in the pyranose ring, and FT-IR spectra also show some changes with irradiation. The dosing differs between solid form and aqueous solution. The κ-, ι-, and λ-carrageenan irradiated at 100 kGy in powder maintained their functional groups, whereas more changes in the functional groupings were observed at 1% aqueous solution carrageenan irradiated at increasing doses from 2 to 100 kGy. The increase in acidity generated by free sulfates followed the decreasing trend λ- > ι- > κ-carrageenan, whereas κ- had the highest increase in reducing sugar and iota had the least. The Mw of carrageenan decreased continuously with increasing dose. Complete degradation of carrageenans was achieved at 100 kGy without an undegraded fraction remaining, whereas in enzyme hydrolysis, a resistant fraction remained. Irradiation of carrageenan with low molecular weight required a low temperature, whereas high molecular weight required a high temperature to ensure homogeneous degradation. In a comparative study, Kalitnik and co-authors reported that the highest antiviral activity was found for LMW derivatives of κ- and κ/β-carrageenans produced by mild acid hydrolysis followed by those obtained by free radical depolymerization and enzymatic degradation [108].

With this method, carrageenan could be easily degraded at an ambient temperature without adding any chemical additives. However, issues in terms of the toxicity of products from irradiated κ-carrageenan need detailed characterization [157], and although the antioxidant activities were enhanced, this has not yet been reported for the antiviral activities.

### 7.3. Fractionation and Purification of Carrageenan Oligosaccharides

Shao and co-authors suggested that the higher concentration (EC_50_ = 89.6 μg/mL) needed for carrageenan to attain similar effectiveness compared with Ribavirin (EC_50_ = 8.3 μg/mL) may limit applications, and further separation and purification of certain molecular-weight κ-carrageenan would be needed [1]. When KCl precipitation was used as a fractionation strategy, the crude hot water extract presented higher sulfate content (33%) than both the non-precipitated fraction (13–18%) and the KCl-precipitated fractions (24–29%), the major fractions being 957 kDa [39].

Natural polysaccharide samples may contain other compounds even after the purification processes. Talarico and co-authors observed that both the commercial ι-carrageenan and the 250 kDa purified fraction showed similar antiviral activity against DENV-2 in C6/36 HT, confirming that the inhibition was not due to any smaller molecules [69]. In other cases, impurities could contribute to activity. *Laurencia obtusa* hydroalcoholic extract inhibited HHV-1 and HHV-2 virus replication in Vero cells with EC_50_ 119 and 141 µg/mL and SI values higher than 29 and 42, respectively [15]. The extracts contained near 40% carbohydrate content, and phenolic compounds accounted for 1.2% and could be also responsible for this activity. Other components present were protein and lipids, accounting for 5–7% of the extract.

The different behavior observed could depend on the seaweed. Crude polysaccharide from the aqueous extracts from *G. griffithsiae* were very active against HSV-1 (IC_50_ 0.5–2.5 μg/mL). After fractionation with KCl, a similar level of antiviral activity was retained in the soluble fractions and in the insoluble kappa/iota/nu-carrageenan, whereas from *C. crenulata,* one of the active galactans isolated and further purified showed a lower IC_50_ than the crude [44]. Alternatively, pre-extraction stages with two water extraction stages at 25 °C before hot water extraction (100 °C) of the residue provided 25% extraction yield and showed IC_50_ valued against HSV-2 and DENV-2 virus comparable to or lower than those found for the purified fractions [39]. However, for in vivo application, crude preparations could be more practical, i.e., for topical prophylactic use in a murine model of vaginal infection with HSV-2, strain MS, animals were treated with a crude water extract from *C. crenulata*, which provided a high level of protection [44].

The production of carrageenan oligosaccharides by the methods described in the previous section may generate some impurities or unwanted compounds. Their purification could be achieved with different procedures including solvent precipitation, membrane fractionation, or gel filtration chromatography. Fractionation according to solubility was proposed for recovering concentrated carrageenan solutions, usually with ethanol, potassium chloride, or ammonium sulfate [70,132]. If the purity of the oligosaccharides after the ethanol precipitation or membrane separation is low, gel filtration chromatography can provide more purified fractions, but this approach is more expensive and more difficult to scale up. Table 3 summarizes the effect of extraction and purification methods on the antiviral action of carrageenans from different seaweeds.

### 7.4. Chemical Modifications

Chemical modifications of carrageenans have been reported to modify the anionic properties, which determine their interactions with biological receptors [38]. A review on the usual chemical modification methods can be found in the work of Chen and co-authors, the most usual being sulfation, acylation, and cyclization [29].

#### 7.4.1. Acylation

The acylation reaction consists of the addition of an acyl group, carboxylic acid anhydrides being commonly used. Additionally, the procedure could be catalyzed using acyltransferases. This chemical modification can not only influence the function of different compounds but also different properties as solubility, stability, or cellular target can be altered [158,159]. Yamada and co-authors found that the acylated low-molecular-weight sulfate carrageenans showed a potential anti-HIV activity [70]. They also observed that the solubility in water was directly influenced by the degree of the acylation; high substitution for λ-carrageenan has shown insolubility in water, and the same behavior was observed for κ-carrageenan [70].

#### 7.4.2. Sulfation

Sulfation alteration consists of the introduction of sulfate groups into hydroxyl groups of the polysaccharide [29]. The modification of the sulfation is used to increase the biological activity of the polysaccharide; for this purpose, the sulfate group is added to the glycosyl polysaccharide radical. The three strategies more frequently used for sulfation of polysaccharides include the chlorosulfonic acid–pyridine, sulfuric acid (using a mixture of concentrated sulfuric acid and butanol), and sulfur trioxide–pyridine methods [158]. Sulfated polysaccharides are the most studied polysaccharides, and evidence of some functions such as antibiosis and antiviral have been found [160].

#### 7.4.3. Cyclization

The cyclization reaction of carrageenans consisting of the formation of 3,6-anhydro-α-d-galactose units from α-d-galactose 6-sulfate follows a pseudo first-order kinetics [161]. This reaction is much faster (20–60 times) for carrageenans of the κ-family than for those of the λ-family, since the clustering of the sulfate groups around the hydroxyl on C-3 of the α-unit in λ carrageenans shields it from ionization or polarization, dropping the cyclization reaction rate [162]. Alkaline treatments are commonly used to enhance the carrageenan cyclization reactions, and the ease with which the cyclization takes place for κ-carrageenans suggests that the alkaline industrial treatments could be carried out under milder conditions [163]. Recent studies found hot alkali cyclization to be an adequate treatment for the red macroalgal species *Dictyota pfaffii* and *Dictyota menstrualis*, which exhibited anti-HIV activity with low toxicity and thus are considered promising candidates for drug development [164]. Hans and co-authors stated that the antiviral activity of carrageenan has distinct inhibitory actions on different viruses because of the cyclization, molecular weight, and level of sulfation of these biopolymers [5].

## 8. Future Trends

Seaweed polysaccharides present many advantages as antiviral drugs, such as the relatively low production costs and wide availability, and they are safe, non-toxic, and show good biocompatibility. Carrageenans are sulfated galactans from red algae with the potential to develop anti-viral drugs and can be proposed alone or in combination with existing drugs, a field deserving further study [29]. Carrageenans could be used for the development of novel broad-spectrum antivirals, with minimum resistance and with other interesting activities, such as anti-inflammatory, antioxidant, and immunomodulatory activities, as an alternative to the toxicity and cost of therapeutic agents [13,16]. The major challenges to their development have been described in relation to:

The need to elucidate the diverse and heterogeneous structure, particularly sulfation degree, molecular weight, conformation, and dynamic stereochemistry, which strongly influences the antiviral activity.

The exploration of activity in clinical trials to confirm the results with in vitro or in vivo models [2,26].

Depolymerization and sulfation are successful strategies to enhance antiviral properties [29].

The green synthesis of gel micro/nanoparticulate systems [165] and the development of novel drug-delivery systems [37] and carriers for antivirals and vaccines [17,31]

The utilization of these compounds from seaweeds can be combined with the valorization of other fractions, following the biorefinery approach [166,167].

## Figures and Tables

**Table 1:** Source and content of the different carrageenan types.

Carrageenan Type	Source (Content, % as Dry Weight)	Reference
kappa	*Kappaphycus alvarezii* (34.3%)	[49]
iota	*Eucheuma denticulatum* (35.5–39.7%)	[50]
	*Calliblepharis jubata* (20–40.4%)	[51,52]
lambda	*Halymenia durvillaei* (29.1%)	[53]
kappa/iota	*Mastocarpus papillatus* (5.4%)	[50]
	*Sarcothalia crispate* (5.4–16.7%)	[50]
	*Chondracanthus chamissoi* (13.5%)	[50]
	*Eucheuma isiforme* (20.4%)	[50]
	*Kappaphycus alvarezii* (30.4–75.6%)	[50]
	*Chondrus crispus* (33.8–60%)	[50,52,54]
	*Ahnfeltiopsis devoniensis* (7.5–30%)	[51,54]
	*Gymnogongrus crenulatus* (12%)	[51]
	*Chondracanthus teedei* (30%)	[51]
	*Gigartina pistillata* (38–58.5%)	[51,52]
	*Coccotylus trancatus* (10%)	[55]
	*Calliblepharis jubata* (28.4%)	[52]
	*Mastocarpus stellatus* (20–45%)	[56]
	*Gigartina skotbbergi* (44.3–50.2%)	[57,58]
	*Gymnogongrus crenulatus* (23.3%)	[52]
kappa/beta	*Betaphycus gelatinum* (71%)	[50]
	*Furcellaria lumbricalis* (69%)	[55]
xi/theta	*Chondracanthus chamissoi* (24.6%)	[50]
	*Chondracanthus teedei* (58%)	[52]
xi/lambda	*Gigartina pistillata* (57%)	[52]
kappa/theta/xi	*Chondracanthus acicularis* (43%)	[51]

**Table 2:** Antiviral activity of commercial or purified carrageenans.

Carrageenan Type	**Chemical Characteristics**	**I** **nhibitory Conc. 50 for Virus and Cell Line (EC_50_, ED_50_, IC_50_)**	**Reference**
ι-carrageenan (commercial)	MW = 500 kDa	HSV-1_Vero_ = 2 µg/mL ASF_Vero_ = 10 µg/mL; ASF_DX Sulf 5000_ = 20 µg/mL EMC_Vero_ = 10 µg/mL; EMC_DX Sulf 5000_ = 5 µg/mL SFV_Vero_ = 10 µg/mL; SFV_DX Sulf 5000_ ≥ 200 µg/mL VSV_Vero_ > 200 µg/mL; VSV_DX Sulf 5000_ ≥ 200 µg/mL Vaccinia_HeLa_ 10 µg/mL Polio type 1_HeLa_ > 200 µg/mL AdV5_HeLa_ > 200 µg/mL	[62]
ι-carrageenan (commercial)	Purity > 95% MW > 100 kDa	IAV_MDCK_ = 0.04–0.20 μg/mL (MOI = 0.01)	[78]
ι-carrageenan (commercial)		DENV-2_Vero_ = 0.39 μg/mL	[79]
ι-carrageenan (commercial)		DENV1_Vero_ = 40.7 μg/mL DENV2_Vero_ = 0.4 μg/mL DENV2_Vero_ = 0.40 μg/mL DENV2_HepG2_ = 0.14 μg/mL DENV3_Vero_ = 4.1 μg/mL DENV3_Vero_ = 1.1 μg/mL DENV3_HepG2_ = 0.63 μg/mL DENV4_Vero_ = 8.2 μg/mL	[8]
ι-carrageenan		HPV_HeLa_ = 1–10 μg/mL	[77]
ι-carrageenan		IAV_MDCK_ = 0.04 µg/mL	[78]
λ-carrageenan (commercial)		DENV1_Vero_ > 50 μg/mL DENV2_Vero_ = 0.15 μg/mL DENV3_Vero_ = 2.0 μg/mL DENV4_Vero_ = 4.2 μg/mL DENV2_Vero_ = 0.22μg/mL DENV2_HepG2_ = 0.14 μg/mL DENV3_Vero_ = 0.60 μg/mL DENV3_HepG2_ = 0.63 μg/mL	[8]
λ-carrageenan (commercial)		HSV-1_PRK_ = 1.6 µg/mL HSV-2_PRK_ = 1.5 µg/mL CMV_HeLa_ = 0.3 µg/mL	[63]
λ-carrageenan		HsV-2_HeLa_ ≤ 7.0 mg/mL	[80]
λ-carrageenan	4 kDa	RABV_HEK-293T_ = 15.9 μg/mL RABV_SK-N-SH_ = 19.9 μg/mL RABV_NA_ = 22.1 μg/mL RABV_BSR_ = 57.7 μg/mL	[46]
κ-carrageenan (commercial)		EEV 71_Vero_ > 10 µg/mL	[24]
κ-carrageenan (commercial)		IAV_MDCK_ = 89.6 μg/mL Ribavirin = 8.3 μg/mL	[1]
κ-carrageenan (commercial)	Purity > 95% MW > 100 kDa	IAV_MDCK_ = 2.70 μg/mL (MOI = 0.01) IAV_MDCK_ = 0.30 μg/mL (MOI = 0.01)	[78]
κ-carrageenan (commercial)		DENV1_Vero_ > 50 μg/mL DENV2_Vero_ = 1.8 μg/mL DENV3_Vero_ = 6.3 μg/mL DENV4_Vero_ > 50 μg/mL	[8]
κ-carrageenan	CO-1, ∼2 kDa CO-2, ∼3 kDa CO-3, ∼5 kDa	IAV_MDCK_ = 32.1 (MOI = 1.0), 42.8 (MOI = 1.0) IAV_MDCK_ = 239 (MOI = 1.0) IAV_MDCK_ = 519 (MOI = 1.0)	[81]
κ-carrageenan (commercial)	KCO-2 kDa, 10.5% sulf KCO-1 kDa, 10.0% sulf KCO-4 kDa, 9.5% sulf KCO-2 kDa, 30.0% sulf KCO-S 2 kDa, 20.0% sulf KCO-DS-2 kDa, 4.0% sulf	IAV_MDCK_ = 128.3 μM IAV_MDCK_ = 14.9 μM IAV_MDCK_ = 141.8 μM IAV_MDCK_ = 23.8 μM IAV_MDCK_ = 41.7 μM IAV_MDCK_ = 137.5 μM	[82]

HSV-1: Herpes simplex virus type 1; ASF: African swine fever virus; EMC: encephalomyocarditis virus; SFV: Semliki Forest virus; VSV: Vesicular stomatitis virus; AdV5: Adenenovirus type 5; IAV: Influenza A virus; DENV-1: Dengue virus type 1; DENV-2: Dengue virus type 2; DENV-3: Dengue virus type 3; DENV-4: Dengue virus type 4; HSV-2: Herpes simplex virus type 2; RABV: rabies virus; HIV-1: Human immunodeficiency virus; DX: Dextran; MOI: Multiplicity of infection; HPV: Human papilloma virus; CO: Carrageenan oligomers; KCO: κ-Carrageenan oligosaccharides; KCO-S: κ-Carrageenan oligosaccharides sulfated derivatives; KCO-DS: κ-Carrageenan desulfated oligosaccharides; BSR: Clone of Baby Hamster Kidney-21 cells; MDCK: Madin–Darby canine kidney cells; HEK: Human embryonic kidney; Vero: African green monkey kidney cells, PRK: Primary rabbit kidney; SK-N-SH; Human neuroblastoma; NA: Mouse neuroblastoma cells.

**Table 3:** Antiviral activity of carrageenan (CG) extracted from red seaweeds.

Carrageenan Source	Extraction, Purification Method	Chemical Characteristics	Inhibitory Concentration 50, Virus, CG Type, Cells = μg/mL or μM	Reference
*Acanthophora specifira*	E: hot W, EtOH pptn	λ-carrageenan	HSV-1_Vero_ = 80.5; RVFV = 75.8	[67]
*Chondrus armatus Tichocarpus crinitus*	E: *Pseudoalteromonas carrageenovora*, 0.2% in 0.1M NaCl + 10 μg enzyme/mL, 37 °C, 24 h, F; FD; GPC (Bio-gel P6)	k- carrageenan, 2.2 kDa κ/β-carrageenan, 4.3 kDa	NA *	[108]
*Cryptonemia crenulata*	E1: W, LSR 1.5, 25 °C, 5 h (C1) E2: 0.025M NaH_2_PO_4_, 80 °C, 6 h (C2) F: KCl frtn, 1.0 M KCl (C2S3) AEC: DEAE-Sephacel, W, 0.25 M NaCl	κ/ι/ν-carrageenan C1: Carb:Sulf:prot = 55.2:33.1:3.6% C2S3: Carb:Sulf:prot = 61.0:28.3:0.5%	HSV-1_C1,Vero_ = 0.5; HSV-2 = 1.1 HSV-1_C2S3,Vero_ = 0.5; HSV-2 = 1.9 HSV-1_Heparin,Vero_ = 3.0; HSV-2 = 1.8 HSV-1_DS8000,Vero_ = 2.8; HSV-2 = 2.5	[44]
*Cryptonemia crenulata*	E: W, 25 °C (C1); 80 ºC (C2, C3) P: KCl gradient pptn. (C1S, C2S) C2S on DEAE-Sephacel (C2S-1-4) C2S-2 on DEAE-Sephacel (C2S-2d)	κ/ι/ν-carrageenan	HSV-1_C3,Vero_ = 1.0 HSV-1_C1S,Vero_ = 0.8 HSV-1_C2-S3,Vero_ = 0.5 HSV-1_2S-2d,Vero_ = 1.0	[44]
*Gigartina atropurpurea* Ts	E: 0.05 M NaHCO_3_, LSR 60, 90 °C, 2 h F; D; FD	λ-carrageenan	HSV-1_k-C,HFF_ = 36; HSV-2 = 1 HSV-1_L-C,HFF_ = 1.5; HSV-2 = 36 HSV-1_ACV,HFF_ = 0.3; HSV-2 = 0.4	[23]
*Gigartina skottsbergii* Ts	E: W, room temp, 3 vol EtOH pptn F: 0.60–0.70 M KCl (T1) Cyclization: NaBH_4_, room temp, 24 h; 3 M NaOH, 80 °C. F: 0.2 M KCl (soluble fract, T1c); D, FD	λ-carrageenan	HSV-1_T1,Vero_ = 0.4; HSV-1_PH_ = 0.8 HSV-1_DS8000,Vero_ = 1.8; HSV-1_PH_ = 0.8	[42]
*Gigartina skottsbergii* Ts	E: W, room temp, KCl pptn (0.65 M)	λ-carrageenan	BoHV-1_MDBK_ = 1.37; SuHV-1 = 73.54	[13]
*Gigartina skottsbergii,* Cs	E: W, room temp; EtOH pptn F: 0.3 M KCl pptn (C1); soluble 0.2 M (C3)	C1: κ/ι-carrageenan C3: μ/ν-carrageenan	HSV-1_k,i-C,Vero_ = 3.2; HSV-1_PH_ = 3.3 HSV-1_m,n-C,Vero_ = 0.9; HSV-1_PH_ = 0.8 HSV-1_DS8000,Vero_ = 1.8; HSV-1_PH_ = 0.8	[42,64]
*Gigartina skottsbergii* Cs	E: W, room temp.; 3 vol. isopropanol pptn.; 0.01–0.10 M KCI pptn; FD	κ/ι-carrageenan; 75–124 kDa Sulf:3,6-AnGal:β-Gal 2S = 33:43:38%	HSV-1_Vero_ = 3.2–4.1; HSV-2 = 1.6–2.3 HSV-1_DS8000,Vero_ = 1.0; HSV-2 = 2.1 HSV-1_Heparin,Vero_ = 1.3; HSV-2 = 2.1	[64]
*Gymnogongrus griffithsiae*	E: W, 100 °C, 2 h (G3: crude extract) P: GC: 2 M KCl (G3S); 1.2 M KCl (G3d); 2 M NaCl (G3S-6); 1M NaCl (G2S-2d)	κ/ι-carrageenan	HSV-1_G3,Vero_ = 0.6 HSV-1_G3S,Vero_ = 2.8 HSV-1_G3d,Vero_ = 1.0 HSV-1_G3S-6,Vero_ = 2.0 HSV-1_G2S-2d,Vero_ = 1.0	[44]
*Gymnogongrus griffithsiae*	E1, E2: W, 25 °C, LSR 3, 16 h, 2 stg; D, FD. Residue, W, 100 °C, 2 h, D, FD (G3) F: KCl frtn, 1.2 M KCl (G3d) AEC: DEAE-Sephacel, W, 0.25 M NaCl	ι/ν/κ-carrageenan, 845 kDa G3: Carb:Sulf:prot = 55.2:33.1:3.6% G3d: Carb:Sulf:prot = 52.0:29.4:1.7% ι- (70%), υ- (17%) and κ- (13%)	HSV-1_G3,Vero_ = 1.1; HSV-2 = 1.2 HSV-1_G3d,Vero_ = 1.0; HSV-2 = 1.0 HSV-1_Heparin,Vero_ = 3.0; HSV-2 = 1.8 HSV-1_DS8000,Vero_ = 2.8; HSV-2 = 2.5	[44]
*Hypnea musciformis*	E: hot W, 0.125 M KCl pptn, D, FD (KC) Full/partial oxidation: primary alcohol, 5 h, NaClO, NaBr; N, D (OKC)	κ-carrageenan, Sigma–Aldrich, 215 kDa KC: Gal:GalA:AnGal:SO_4_ = 1.0:0.0:1.0:1.2 OKC: 163 kDa, Gal:GalA:AnGal:SO_4_ = 0.33:0.67:0.81:1.08	HSV-1_KC,Vero_ = 13.8; HSV-2 = 11.0 HSV-1_OKC,Vero_ = 1.7; HSV-2 = 0.98	[38]
*Hypnea musciformis*	E: hot W, 0.125 M KCl pptn, D, FD (KC) Full/partial oxidation: primary alcohol, 5 h, NaClO, NaBr; N, D (OKC)	ι-carrageenan, Sigma–Aldrich, 460 kDa IC: Gal:GalA:AnGal:SO_4_ = 0.97:0.03:1.0:2.0 OIC: 324 kDa, Gal:GalA:AnGal:SO_4_ = 0.33:0.67:0.81:1.1	HSV-1_KC,Vero_ = 0.67; HSV-2 = 0.43 HSV-1_OKC,Vero_ = 0.40; HSV-2 = 0.40	[38]
*Meristiella gelidium*	E: W, 25 °C (E1); 100 °C (E2) F: KCl pptn (E2F)	ι/κ/ν-carrageenan E2F: 957 kDa, Carb:sulf:prot = 43:29:9%	HSV-2_E1,Vero_ = 0.06; C6/36 DENV-2_HT_ = 0.79 HSV-2_E2__,__Vero_ = 0.05; C6/36 DENV-2_HT_ = 0.14 HSV-2_E2F,Vero_ = 0.04; C6/36 DENV-2_HT_ = 0.21 HSV-2_Heparin,Vero_ = 0.6; C6/36 DENV-2_HT_ = 1.9 HSV-2_DS8000,Vero_ = 0.6; C6/36 DENV-2_HT_ = 0.9	[39]
*Kappaphycus alvarezii*	E: 0.1 N HCl; LSR 100, 60 °C, 4 h + 37 °C, 24 h neutral. (0.1M NaOH); EtOH pptn F; FD; GPC (Bio-gel P6)	κ-carrageenan, Sigma, 1.2–3.0 kDa	NA *	[108]
*Plocamium cartilagineum*	E: 0.05 M NaHCO_3_, LSR 60, 90 °C, 2 h F; D; FD	Complex sulfated galactan	HSV-1_k-C,HFF_ = 5.4; HSV-2 = 36	[23]
*Schizymenia pacifica*	E: 20% citrate-phosphate, 4 °C, 16 h P: DEAE-cellulose chromatography, eluted with NaCI, and Sepharose CL-4B	λ-carrageenan; 2 × 10^3^ kDa Gal:Sulf:3,6-AG = 73:20:0.65	HIV_MT-4_ = 9.5 × 10^3^ IU/mL	[45]
*Solieria chordalis*	CE: W, room temp, 12 h, 1% KOH (E1); 85 °C, 3 h (E2) F: EtOH pptn + sodium acetate MAE1: 90 °C, MAE2: 105 °C; 25 min	ι-carrageenan CE: Carb:Sulf:prot = 15.4:5.0:3.0 MAE1: Carb:Sulf:prot = 13.5:5.1:2.1 MAE2: Carb:Sulf:prot = 13.5:4.7:2.3	HSV-1_CE_ = 0.1; _Acyclovir_ = 0.2 HSV-1_MAE_ = 0.3–0.5; _Acyclovir_ = 0.5	[66]

E: Extraction; F: Filtration; frtn: Fractionation; Carb: Carbohydrate; C: Centrifugation; CE: Conventional extraction; Cs: Cystocarpic stage; D: dialized; Gal: Galactose; GC: Gel chromatography; GPC: Gel permeation chromatography; LSR: Liquid solid ratio; MAE: Microwave assisted; D: Depolymerization; neutral.: Neutralization; HFF: human foreskin fibroblast; HT: human B cell lymphoma; MT-4: human T cell leukaemia; pptn: Precipitation; P: Purification PH: Human diploid foreskin fibroblast cell line; Prot: Protein; room temp: Room temperature; stg: Stages; Sulf: Sulfate; Ts: Tetrasporic stage; Vero: Vero cells; Virus: DENV: Dengue; HSV: Herpes simple; Bo-HV: Bovine Herpes; Su-HV: Suid Herpes; RVFV: Rift Valle Fever; W: Water; NA, not applicable, assays performed in *Nicotiana tabacum* leaves.

## Data Availability

Data found in the referenced sources.
